# Associated Proteins’ Proteomic Profiling of Equine Periodontal Ligament Stem Cell–Derived Exosomes for Diabetic Wound Healing Regeneration

**DOI:** 10.1155/vmi/4223013

**Published:** 2026-10-23

**Authors:** Ruchianasari Deliaputri, Teguh Budipitojo, Aris Haryanto, Medania Purwaningrum

**Affiliations:** ^1^ Master Program of Veterinary Science Faculty of Veterinary Medicine Universitas Gadjah Mada Yogyakarta 55281 Indonesia ugm.ac.id; ^2^ Department of Anatomy Faculty of Veterinary Medicine Universitas Gadjah Mada Yogyakarta 55281 Indonesia ugm.ac.id; ^3^ Department of Biochemistry and Molecular Biology Faculty of Veterinary Medicine Universitas Gadjah Mada Yogyakarta 55281 Indonesia ugm.ac.id

**Keywords:** diabetic ulcer, equine periodontal ligament stem cells, exosomes, proteomics, wound healing

## Abstract

**Background and Aim:**

Exosome‐based cell‐free therapies have shown significant potential to enhance diabetic ulcer healing. Exosomes derived from equine periodontal ligament stem cells (ePDLSCs) offer a viable alternative for regenerative wound‐healing treatments. This study aimed to characterize and analyze the protein composition of these exosomes and assess their efficacy in treating diabetic wounds in vivo.

**Materials and Methods:**

This study employed RT‐qPCR and Nanoparticle Tracking Analysis (NTA) to isolate and characterize ePDLSCs–exosomes, western blot analysis of ePDLSCs–exosomes, proteomic protein profile analysis of ePDLSCs–exosomes using high‐resolution mass spectrometry (HRMS), western blot analysis for ACTB and VIM, potential protein profiling with proteomic analysis data associated with wound healing (Reactome, gene ontology [GO], KEGG pathways analysis), and assessment of ePDLSCs–exosome gel in vivo wound healing of diabetic ulcer wounds.

**Results:**

The findings of this study reveal that exosomes derived from ePDLSCs, as shown by NTA, had a size distribution of approximately 114 ± 39 nm and expressed the exosome surface marker CD9. ePDLSCs–exo contained 35 proteins with potential involvement in wound healing, with the largest PSMs being annexin 2 (ANXA2), actin beta (ACTB), and vimentin (VIM), as shown in Western blot analysis. Functional enrichment analysis revealed the potential biological significance and signaling pathways of proteins supporting wound healing identified from ePDLSCs–exo. Furthermore, in vivo experiments showed ePDLSCs–exosomes impacted diabetic wound healing, with higher healing rates in the exosome‐treated group.

**Conclusion:**

Exosomes derived from ePDLSCs have demonstrated regenerative and healing properties, particularly in the context of tissue damage associated with diabetes.

## 1. Introduction

In regenerative therapies, equine mesenchymal stem cells (MSCs) have been extensively studied because of their multipotent capabilities and immunomodulatory characteristics. Equine MSCs are promising candidates for regenerative therapies, especially in wound‐healing applications [1–3]. Notably, dental stem cells (DSCs) [4] are a subset of equine MSCs that offer potential for regenerative treatments utilizing less invasive methods, including periodontal ligament stem cells (PDLSCs) [5]. Equine periodontal ligament cells meet MSC criteria by adhering to plastic, forming fibroblast‐like colonies [6], expressing CD29 [7], CD90, CD73, and CD105, lacking CD18 and CD45, and differentiating into osteogenic, chondrogenic, and adipogenic cells [7–9]. COL1 and COL3 expression in equine periodontal ligament (PDL) cells is significant, indicating enhanced collagen production and turnover. This activity facilitates the remodeling process during wound healing [10].

Cell therapy with MSCs has high regenerative potential but disadvantages, including reduced definitive differentiation with proliferation and aging during in vitro culture, which affect MSC regulatory and therapeutic ability [6]. Cell‐free therapy is an alternative that utilizes MSC paracrine secretion [11], such as conditioned medium (CM), containing growth factors and proteins involved in tissue regeneration [12, 13]. Extracellular vesicles (EVs), divided into microvesicles (MVs) and exosomes (EXs), are part of CM and transfer proteins and genetic material in cell‐free therapy [14]. EXs are widely used because they offer low immunogenicity and tumorigenicity, minimal ethical concerns, and high stability and can be loaded with drugs and transported to target cells [15, 16].

Previous studies reported that equine MSC‐EVs lower inflammation [17, 18]. Equine MSC EXs, a subtype of MSC‐EVs, also meet the criteria, as NTA analysis shows a size range of 35–95 nm [19]. Although explored as regenerative therapy, few studies explain their potential, making them an intriguing therapeutic option for further study in degenerative diseases.

Degenerative diseases reduce the quality of life by impairing organ function. They are a concern, including diabetes mellitus, whose cases are rising. Diabetes mellitus (DM) cases are predicted to reach 853,000 by 2050 (International Diabetes Federation, 2025). In animals, diabetes mellitus has been reported in 1072 cases in dogs [20], 1053 in cats [21], and horses with equine metabolic syndrome (EMS) can develop insulin resistance linked to Type 2 diabetes [22–24]. DM increases the risk of diabetic wounds, which heal slowly because of dysregulated, prolonged M1 macrophage (proinflammatory) polarization. Normally, wounds shift to M2 macrophages (prohealing) around the third day after injury [25, 26]. It occurs in dogs [27], cats [28, 29], horses with EMS‐related laminitis [30, 31], and humans [32].

The incidence of diabetic wounds continues to increase throughout the year, necessitating the development of alternative treatments in addition to wound dressing treatments. The presence of various bioactive components in equine MSC‐eV that support tissue regeneration makes EXs from ePDLSCs a promising alternative treatment for diabetic ulcers, which was a key focus of this study. Based on this background, we aimed to investigate the potential of ePDLSCs EXs in diabetic wound‐healing therapy as a translational medicine.

## 2. Methods

### 2.1. Equine Periodontal Ligament Stem Cell (ePDLSC)–EX Preparation

The ePDLSCs–EXs used in this study were derived from ePDLSCs. The isolation protocol, derived from research, utilized the tissue explant method [33]. The ePDLSCs used in this study were passage 3–5 cells, characterized according to the requirements for MSCs in our previous study [7]. CM was obtained from ePDLSCs and collected for EX isolation. ePDLSCs–EX isolation was performed using Total Exosomes Isolation reagent (Invitrogen) following the product’s protocol, as previously reported in MSC‐derived EXs studies [34, 35]. ePDLSC EXs were characterized for size and concentration using Nanoparticle Tracking Analysis (NTA) ViewSizer 3000 (HORIBA Scientific) and for the marker CD9, determined using RT‐qPCR in our study.

### 2.2. Profile Protein ePDLSCs With Proteomics

Protein profile analysis was performed by high‐resolution mass spectrometry (HRMS). We analyzed EXs from ePDLSCs using a Thermo Scientific Orbitrap Exploris 240 HRMS (Bremen, Germany). The protein profiles obtained were examined with the online resource database Reactome (https://reactome.org/), and proteins associated with wound healing were displayed in a heatmap and Venn diagram. Functional enrichment analysis was performed using the STRING database (https://string-db.org/) to investigate the biological significance of proteins identified from ePDLSCs–exo. Gene ontology (GO) enrichment analysis was conducted to classify proteins into biological process (BP), molecular function (MF), and cellular component (CC) categories. Kyoto Encyclopedia of Genes and Genomes (KEGG) pathway analysis was subsequently performed to identify significantly enriched signaling pathways associated with the protein dataset. Statistical significance was evaluated using the false discovery rate (FDR) adjusted *p* values provided by STRING, and enriched terms with FDR *p* value < 0.05 were considered significant. The top enriched GO terms and KEGG pathways were selected based on enrichment significance and biological potential relevance to wound healing.

### 2.3. Western Blot Analysis

Western blot analysis was performed to assess protein expression in exosomal samples derived from ePDLSCs, following the manufacturer’s protocols and a previously reported workflow [36] (Invitrogen, USA). Exosomal protein concentration was determined using the BCA Protein Assay Kit (Thermo Fisher Scientific, USA); samples were lysed in a buffer containing protease inhibitors and quantified, and 60 µL of protein was denatured. These proteins were separated using a Bolt 4%–12% Bis‐Tris, 1.0 mm, Mini Protein Gel (Invitrogen, USA) and subsequently transferred to a polyvinylidene difluoride (PVDF) membrane via the iBlot dry blotting system (Invitrogen, USA). The membranes were processed using the iBind Western System (Invitrogen, USA) and incubated with primary antibodies against β‐actin (ACTB) and vimentin (VIM). ACTB and VIM were selected as a reference protein and a target of interest due to their abundance in exosomal cargo, as indicated by previous proteomic analysis. Protein bands were visualized using a chemiluminescence (ECL) detection system, captured with the iBright Imaging System (Invitrogen, USA).

### 2.4. Manufacture and Evaluation of ePDLSCs–Exo Gel

A wound‐healing test for diabetic ulcers was performed using a gel base as an intermediate for ePDLSCs–EXs. The gel underwent pharmaceutical evaluation, including organoleptic, dispersion, adhesion, viscosity, and pH testing. The preparation of the ePDLSCs–EX gel conformed to pharmaceutical testing standards with the same protocol in our previous study [37]. Calculations were conducted for each wound application over 14 days, followed by ongoing testing on rat test subjects.

### 2.5. In Vivo Evaluation of ePDLSC–EX Gel

#### 2.5.1. Experimental Animal

Animal handling and research procedures followed ethical approval from the Ethics Committee, Faculty of Veterinary Medicine, UGM, Yogyakarta, Indonesia (Approval No. 57/EC‐FKH/int./2025). The sample size was determined a priori using G∗Power 3.1.9.7 software (Heinrich Heine University, Düsseldorf, Germany). The calculation resulted in a minimum of four animals per group; therefore, 16 rats were included in the four experimental groups. Wistar rats (*Rattus norvegicus*) were housed in the Nutrition Laboratory, Center for Food and Nutrition Studies, Universitas Gadjah Mada. Male Wistar rats, 8 weeks old and weighing 135–180 g, were acclimated, given unrestricted basal feed, and randomly divided into four groups (*n* = 4): nondiabetic rats with wounds (normal control), diabetic rats with wounds treated with gel base (DM Base Gel), commercial ointment (DM commercial), and ePDLSCs‐EX gel (DM ePDLSCs‐exo).

#### 2.5.2. Induction of Diabetes and Wound Model Treatment

After acclimatization, rats were fasted for 12 h and injected intraperitoneally with nicotinamide (NA) 110 mg/kg BW and streptozotocin (STZ) 45 mg/kg BW. Blood glucose was measured on Day 3 after injection; rats with fasting blood glucose levels > 250 mg/dL were considered diabetic. Diabetic rats were then anesthetized for biopsy wound creation, underwent a 6‐mm back biopsy with a sterile biopsy punch, and received ePDLSCs–EX gel daily for 14 days.

The wound area was measured on Days 0, 3, 7, 10, and 14 to monitor closure using WHR = (initial measurement of wound area–day N wound area)/initial measurement of wound area × 100%. On Day 14, rats were euthanized for histopathology analysis. Histopathological analysis included epithelial thickness, inflammatory cell infiltration, fibroblast density, and microvessel formation.

### 2.6. Laser Speckle Imaging for Wound Perfusion Assessment

On Day 14, tissue perfusion in experimental rats was measured using laser speckle imaging (RFLSI‐ZW; RWD Life Science, China). Under anesthesia, the wound area was cleaned, and rats were positioned under the RFLSI camera to image wound surfaces. Perfusion data were collected in perfusion units (PUs), visualized on a map, and analyzed using RFLSI software (RWD Life Science, China).

### 2.7. Data Analysis

The study’s findings were examined with SPSS Statistics software Version 26.0 for Windows (IBM Corporation, USA). The wound‐healing assessment was analyzed using the nonparametric Kruskal–Wallis test, followed by a post hoc Mann–Whitney *U* test to compare two independent groups, with *p* < 0.05 considered statistically significant. The data were displayed graphically in GraphPad Prism, Version 10.4.0, for Windows (GraphPad Software, Inc., USA).

## 3. Results

### 3.1. Preparation and Characterization of ePDLSCs–EXs

ePDLSCs were extracted and cultured up to passages 3–5, while EXs were obtained from ePDLSCs‐CM through a sequential centrifugation technique following the Total EXs Isolation (Invitrogen, Catalog No. 4478359) product protocol (Figure 1A). Nanotracking analysis verified the characterization of ePDLSCs–EXs, showing a morphology and particle size between 30 and 200 nm, with an average diameter of 114 ± 39 nm (Figure 1B–D), and the concentration of ePDLSCs–EXs was confirmed (Figure 1E). The characterization of ePDLSCs–EXs confirmed the presence of the exosomal marker CD9 (Figure 1F).

**FIGURE 1:**
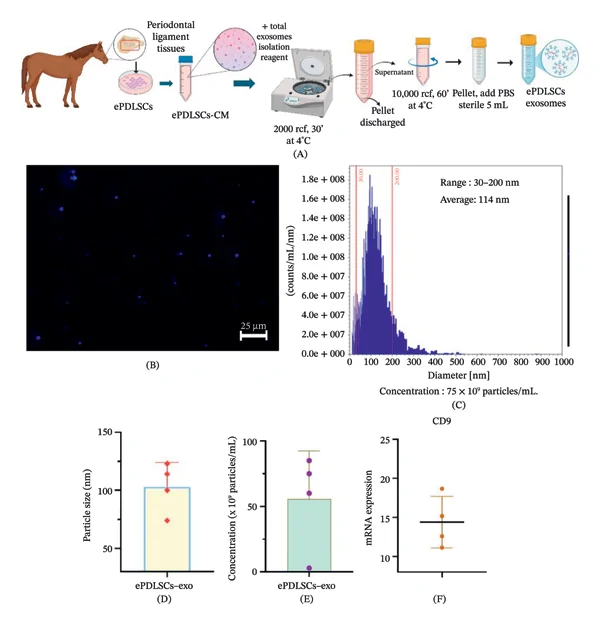
ePDLSC–exosome preparation. (A) ePDLSCs were isolated and cultured, and exosomes were isolated. (B–D) The morphology and size distribution of the exosomes obtained from ePDLSCs were analyzed. (E) The quantity of exosomes derived from ePDLSCs was measured. (F) RT‐qPCR was utilized to assess the mRNA levels of the exosomal surface marker CD9.

### 3.2. Protein Profiling of ePDLSCs–EXs

List of proteins expressed in the EXs of ePDLSCs, as determined by HRMS screening. The heatmap (Figure 2A) visualizes proteins associated with wound healing. We identified 35 proteins with the highest PSM counts involved in wound healing, including anti‐inflammatory proteins, growth factors, and extracellular matrix (ECM)‐remodeling facilitators. Western blot analysis detected ACTB and VIM at their expected molecular weights, with bands at approximately 42 and 55 kDa, respectively. This is consistent with their identification in the proteomic profile (Figure 2B). A Venn diagram from proteomic profiling of ePDLSCs–EX, analyzed using Reactome, was sorted by potential pathway relevance to wound healing (Figure 2C), focusing on ECM organization, Cytokine signaling, VEGF signaling, and PDGF signaling. Functional enrichment analysis showed the top enriched GO terms (Figure 2D) and KEGG pathways ranked by –log10(FDR). The results highlight significant involvement of ECM–receptor interaction, focal adhesion, and PI3K‐Akt signaling pathways potentially in tissue development and wound healing (Figure 2E).

**FIGURE 2:**
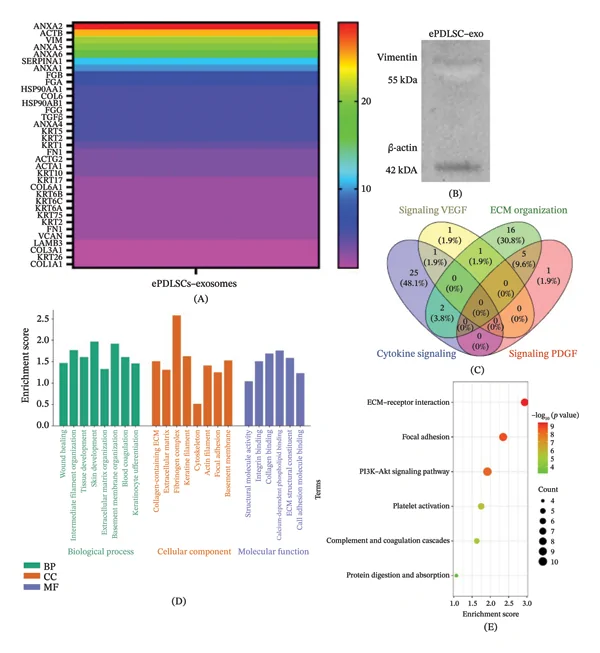
Protein profiling of ePDLSCs‐derived exosomes. (A) Heatmap generated from proteomic data for potential proteins related to wound‐healing therapy. (B) Western blot results with VIM and ACTB detected in ePDLSCs–exo. (C) Signaling pathway from the potential protein related to wound healing. (D) Gene ontology (GO) terms include biological process (BP), cellular component (CC), and molecular function (MF) related to wound healing. (E) KEGG pathway analysis potentially related to wound healing.

### 3.3. The Role of Protein Profiling in Wound Healing Phase

Proteins from ePDLSC‐derived EXs that may play a role in each phase of wound healing (Figure 3A) show that the proteomically analyzed proteins are involved in every stage of wound healing—from hemostasis and inflammation to proliferation and remodeling—thereby accelerating diabetic wound healing. Reactome analysis also identified signaling pathways that may contribute to each phase. KEGG pathway analysis further showed that ePDLSCs‐derived EXs contain proteins involved in ECM–receptor interactions, focal adhesion, PI3K–Akt signaling pathways, platelet activation, the complement and coagulation cascade, and the protein digestion and absorption pathway (Table 1), which may also contribute to diabetic wound healing (Figure 3B–G).

**TABLE 1:** Functional enrichment analysis of potential ePDLSCs–exo proteins associated with wound healing (KEGG pathway).

Pathway	Proteins contributing to pathways	FDR
ECM–receptor interaction	COL6A1, COL6A3, COL6A2, COL1A1, LAMB3, ITGB1, VTN, FN1	5.10E‐10
Focal adhesion	ACTB, COL1A1, COL6A3, COL6A1, COL6A2, LAMB3, ITGB1, VTN, FN1	2.10E‐09
PI3K‐Akt signaling pathway	COL6A3, COL6A1, COL6A2, COL1A1, LAMB3, ITGB1, VTN, FN1, HSP90AA1, HSP90AB1	6.49E‐09
Platelet activation	COL1A1, ACTB, ITGB1, FGA, FGB, FGG	2.23E‐06
Complement and coagulation cascades	PLG, FGB, FGG, FGA,VTN	8.12E‐06
Protein digestion and absorption	COL6A3, COL6A2, COL1A1, COL6A1	0.00064

*Note:* The potential proteins contributing to each enriched term were identified from the input protein list used for enrichment in KEGG pathway analysis (FDR < 0.05).

**FIGURE 3:**
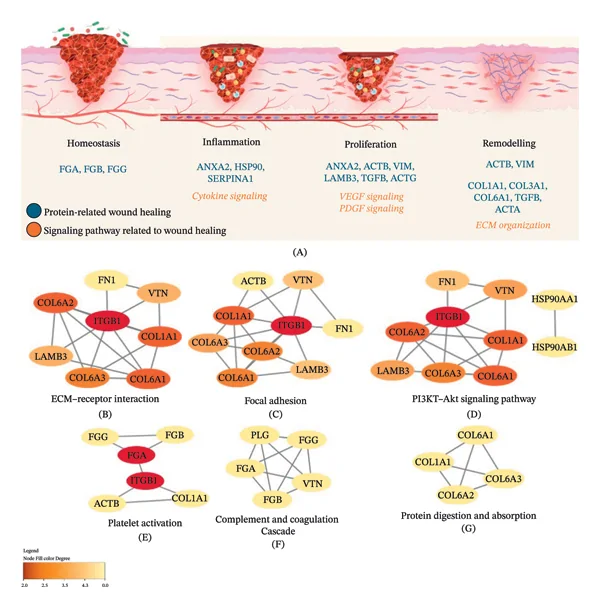
The role of protein profiling in the wound‐healing phase. (A) Illustration of the potential of ePDLSC–exosome proteins and signaling pathways (Reactome analysis) in each phase of wound healing from hemostasis and inflammation to proliferation and remodeling—thereby accelerating diabetic wound healing. Results of analysis of the KEGG pathway and ePDLSC–exosome proteins that play a role in (B) ECM–receptor interactions, (C) focal adhesion, (D) PI3K–Akt signaling pathways, (E) platelet activation, (F) complement and coagulation cascade, and (G) protein digestion and absorption, which may also contribute to diabetic wound healing.

### 3.4. ePDLSCs–EX Gel in Diabetic Wound Healing Evaluation

We performed an in vivo assessment involving four treatment groups (*n* = 4) (Figure 4A). The formulated base gel (Figure 4B) satisfied the essential standards for pharmaceutical gel evaluation. Over 14 days, the in vivo assessment showed a steady increase in wound closure percentage. Macroscopic examination indicated that the gel with EXs from ePDLSCs showed notable effectiveness in healing diabetic wounds, with wounds fully closing by Day 14 (98.81% ± 0.023), unlike the control, DM base gel, and DM commercial groups (Figure 4C). There was a marked difference between the DM base gel and DM ePDLSCs–exo groups (*p* = 0.0286), as well as between the DM commercial and DM ePDLSCs–exo groups (*p* = 0.1000) (Figure 4D). Laser speckle contrast imaging (LSCI) revealed greater perfusion in wounds treated with ePDLSCs–EXs (Figure 4E), evidenced by the increased presence of yellow–red regions on the perfusion map. Laser speckle imaging also assessed local tissue perfusion, and ΔPU values showed a smaller perfusion difference in EX‐treated wounds, suggesting a perfusion profile closer to surrounding normal tissue (Table 2). In the untreated control group, the normal cohort exhibited a ΔPU of −319.19. Conversely, the base gel treatment led to a ΔPU of −130.39, indicating decreased blood flow in untreated wound areas. The commercial drug improved blood flow compared with the control, with a ΔPU of −119.6 in diabetic rats, indicating enhanced vascular function. ePDLSCs–exo treatment showed the greatest improvement, achieving a ΔPU of −55.59 in diabetic rats, nearing normal blood flow levels. These results imply that ePDLSCs–exo treatment can more effectively stimulate neovascularization and improve wound blood flow than commercial therapy or no treatment.

**FIGURE 4:**
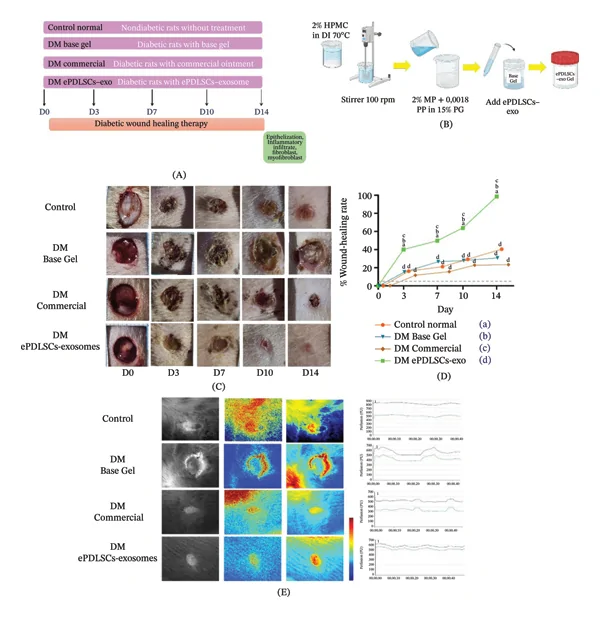
Evaluation of ePDLSC–exosome gel and wound‐healing in vivo assessment. (A) Assessment of the in vivo treatment group (*n* = 4). (B) Formulation and pharmaceutical assessment of ePDLSC–exosome gel. (C) Macroscopic observation of wound closure on Days 0, 3, 7, 10, and 14. (D) Wound‐healing rate percentages on Days 3, 7, 10, and 14. Superscript letters denote significant differences compared to control nondiabetic (a), diabetic base gel (b), commercial diabetic (c), and ePDLSCs–exo diabetic (d) (*p* < 0.05). (E) Examination of wound perfusion on Day 14.

**TABLE 2:** Perfusion unit (ΔPU) measurements were used to describe the local perfusion pattern following wound treatment on the 14th day.

Area treatment group	Nondiabetic normal control	DM base gel	DM commercial	DM ePDLSCs–exo
Normal tissue	511.39	439.89	318.78	510.45
Wound tissue	830.58	570.28	438.38	566.04
Representative ΔPU	−319.19	−130.39	−119.6	−55.59

*Note:* Perfusion units (ΔPU) in the wound area following treatment. ΔPU was calculated as the difference between perfusion in normal adjacent tissue and the wound area.

### 3.5. Histopathology Analysis in Wound Healing Evaluation

Histopathological examination of skin from different treatments showed (Figure 5A) at all three magnifications, indicating better structural completeness with ePDLSCs–exo treatment. Histopathological analysis confirmed these in vivo results, showing that re‐epithelialization was most pronounced in the ePDLSCs–exo group (Figure 5B). Histopathological analysis also revealed that angiogenesis and fibroblast numbers were highest in the ePDLSCs–exo group, with minimal infiltration of inflammatory cells. The infiltration score of inflammatory cells, including neutrophils, was significantly different between ePDLSCs–exo and the control (*p* = 0.040), base gel (*p* = 0.011), and commercial treatments (*p* = 0.040) (Figure 5C). The macrophage infiltration score corroborated these findings, indicating a significant difference between ePDLSCs–exo and the control (*p* = 0.017), base gel (*p* = 0.011), and commercial treatments (*p* = 0.015) (Figure 5D). These results suggest that the ePDLSCs–exo gel facilitates wound healing, outperforming commercial drugs by enhancing healing rates and accelerating wound healing in diabetic wounds through the transition from M1 (proinflammatory) to M2 (anti‐inflammatory) macrophages compared to the control, base gel, and commercial drugs.

**FIGURE 5:**
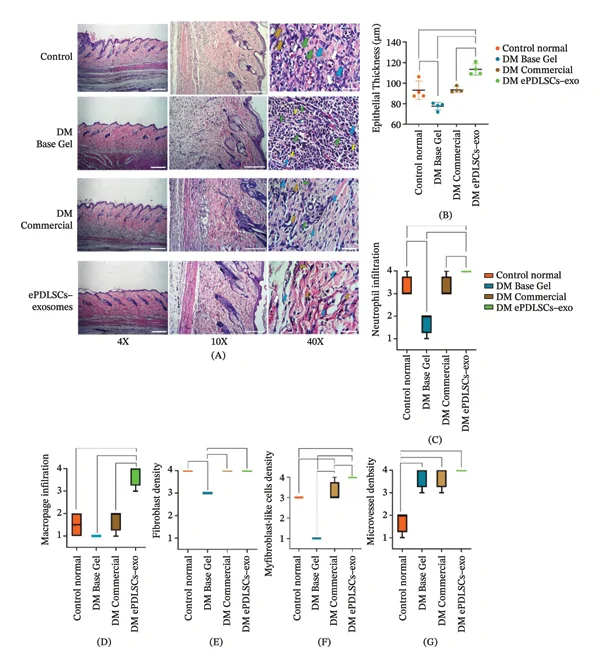
Histopathological analysis of wound healing. (A) Histopathological morphology (yellow arrow: neutrophil, green arrow: macrophage, blue arrow: fibroblast, light brown arrow: myofibroblast‐like cells, white arrow: blood vessels). (B) Histomorphometry of epithelial thickness on Day 14 for each group and treatment. (C, D) Inflammatory infiltration scores. (E) Fibroblast density score. (F) Myofibroblast‐like cell density scores. (G) Microvessel blood score. Boxes represent the interquartile range (IQR). Bars denote significant differences (*p* < 0.05) (scale bar = 25 μm).

The fibroblast count score, crucial for wound closure, exhibited a significant difference between ePDLSCs–exo treatment and base gel (*p* = 0.008) (Figure 5E). This was further supported by the score for myofibroblast‐like cells, which showed a significant difference between ePDLSCs–exo treatment and control (*p* = 0.008), base gel (*p* = 0.008), and commercial treatments (*p* = 0.040) (Figure 5F). These findings suggest that ePDLSCs–exo promotes wound healing by accelerating the growth and remodeling phases, with the ECM playing a crucial role in wound closure. In addition to reducing inflammatory cell infiltration and promoting faster ECM formation, ePDLSCs‐exo also aided blood vessel maturation, as shown by larger lumens and thicker vessel walls (white arrows). The microvessel count score also differed significantly between ePDLSCs–exo treatments and the control (*p* = 0.011) (Figure 5G). In contrast, the diabetic rats exhibited no significant differences between treatments, as the number of microvessels remained insufficient in the base gel treatment. This insufficiency suggests incomplete formation, indicating that the condition was still in the inflammatory phase on Day 14. Table 3 lists the scoring parameters.

**TABLE 3:** Scoring determination in histopathological analysis.

Parameter/score	1	2	3	4
Neutrophil infiltration	> 15	11–14	6–10	1–5
Macrophage infiltration	> 15	11–14	6–10	1–5
Fibroblast density	< 5	5–9	10–19	> 20
Myofibroblast‐like cells density	< 5	5–9	10–19	> 20
Microvessel density	< 15	11–15	5–10	< 5

*Note:* Scoring parameters used in the histopathological analysis in this study.

## 4. Discussion

Diabetic wounds remain a clinical challenge because of multifactorial disruptions in the healing cascade [38]. This study highlights the potential of the evaluated treatments to promote tissue regeneration and suggests translational strategies for chronic wounds. Therapeutic applications for degenerative diseases, including diabetic wounds, leverage EXs, and their paracrine capabilities in wound healing [39, 40]. MSC‐derived EXs have shown superior therapeutic potential versus conventional therapies [41–43].

In earlier in vitro studies, we effectively isolated and characterized ePDLSCs, showcasing their potential as an alternative therapeutic strategy in bone tissue engineering (BTE) and highlighting their promise in wound‐healing treatments [7]. Therefore, this study aimed to explore the potential of ePDLSCs‐derived EXs as a cell‐free therapy for diabetic wound healing and to profile the potential proteins involved in this process.

Given the quantity of EX particles and proteins in ePDLSCs–EXs, we conducted in vivo rat assessments to evaluate their efficacy for diabetic wounds. The results from the pharmaceutical gel evaluation and rat application showed complete macroscopic wound closure by Day 14. Histomorphometric analysis showed that ePDLSCs–exo administration improved tissue regeneration, as indicated by improved re‐epithelialization, more mature granulation tissue formation, and better tissue organization compared with the other groups. In addition, the number of inflammatory cells in the wound area also appeared reduced in the ePDLSCs–exo treatment group, indicating an improved inflammatory response during wound healing.

This tissue repair is associated with the potential bioactive protein content in ePDLSCs–exo. Proteomic analysis identified candidate proteins such as ANXA2, ACTB, and VIM as the most prevalent. These proteins are recognized for their roles in cell migration, cytoskeletal rearrangement, and tissue regeneration, which are crucial processes during re‐epithelialization and new tissue formation [44]. GO enrichment analysis corroborated these findings, showing enrichment in BPs such as wound healing, skin development, tissue development, and keratinocyte differentiation. Additionally, Reactome analysis revealed enrichment in the cytokine signaling pathway, which is important for regulating communication among immune cells and the inflammatory response during wound healing [45]. Previous studies have shown that CM derived from adipose‐derived mesenchymal stem cells (AD‐MSCs) can reduce inflammation by blocking the mRNA transcription of proinflammatory cytokines and chemokines [46]. Consequently, the histopathologically observed reduction in inflammatory cell infiltration may suggest that ePDLSCs–exo helps modulate the inflammatory environment in diabetic wounds. This modulation may accelerate wound healing by facilitating a faster transition from M1 (proinflammatory) to M2 (anti‐inflammatory) macrophages, thereby shortening the inflammatory phase of wound healing [47–49]. Previous studies have demonstrated that MSC‐derived EXs can exert immunomodulatory effects by attenuating inflammatory cytokine responses, inhibiting complement activation, and modulating neutrophil‐associated inflammatory processes. In particular, MSC‐derived EXs have been reported to reduce IL‐17–associated inflammation and inhibit formation of the terminal complement complex C5b‐9 in inflammatory skin lesions [50]. These observations provide a potential context for interpreting the present proteomic findings, in which ePDLSC‐derived EXs were associated with BPs involving the acute‐phase response, complement and coagulation cascades, and neutrophil‐related pathways.

Enrichment in these processes is consistent with the increased re‐epithelialization observed in histomorphometric analysis, which revealed better epithelial thickness in the ePDLSCs–exo group, with a complete epithelial structure, indicating the support of various ePDLSCs–exo proteins in epithelial tissue regeneration, such as ECM proteins, which are involved in the transition from the proliferation phase to the remodeling phase [51]. In addition, the GO results also indicate enrichment in ECM organization, collagen binding, and integrin binding. These findings are consistent with increased ECM deposition in wound tissue, as ECM remodeling is a critical stage in granulation tissue formation and skin regeneration.

In addition to repairing tissue structure, ePDLSCs–exo also enhances perfusion in the wound area. Wound perfusion assessments corroborated these findings, showing that rats treated with the ePDLSCs–exo gel had higher average wound perfusion values. The perfusion pattern in EX‐treated wounds was consistent with the wound‐healing progression observed in the macroscopic and histopathological assessments. This enhancement was accompanied by mature vascular structures, as evidenced by enlarged lumens and thickened vessel walls in H&E staining. These observations are consistent with the proteomic profile of ePDLSCs–exo, which includes potential angiogenic proteins such as ANXA2, known to facilitate endothelial activation and vascular maturation [52]. Reactome analysis also showed enrichment in pathways potentially involved in VEGF signaling, which plays a key role in angiogenesis. Activation of this pathway can contribute to new blood vessel formation, thereby increasing the supply of oxygen and nutrients needed during wound healing. Collectively, improved perfusion, the presence of mature vessels, and abundant proangiogenic proteins suggest that ePDLSCs–exo promotes vascular remodeling and accelerates diabetic wound healing through paracrine angiogenic mechanisms [53].

Histopathological analysis also showed improved numbers of fibroblasts and myofibroblast‐like cells. Comparison of fibroblast and myofibroblast‐like cell counts in the ePDLSCs–exo‐treated groups indicated a well‐progressed transition from the proliferation phase to the remodeling phase. The potentially involved enrichment in the PDGF signaling pathway also supports these histopathological findings, as PDGF is known to play a role in fibroblast recruitment, collagen synthesis, and granulation tissue formation [54]. The ECM–receptor interaction and focal adhesion pathways identified through KEGG analysis indicate communication between cells and the ECM, which is necessary for keratinocyte and fibroblast migration during wound closure. Complete wound closure by Day 14 in groups treated with ePDLSCs–exo was also supported by the identification of fibrinogen proteins (FGA, FGB, and FGG) highly expressed by ePDLSCs–exo; these candidate proteins support their role in forming the blood clot network during the hemostasis phase of wound healing [55, 56]. Fibrinogen proteins (*α*, β, and *γ*) have also been shown to be expressed during the acute phase based on equine proteome data [57].

The remodeling phase of wound healing is supported by ECM formation, the final process of macroscopic and microscopic wound closure. Complete wound closure in the ePDLSCs–exo treatment group was supported by potential proteins such as COL1A1, COL3A1, and COL6A1 in scar tissue formation [58, 59]. This aligns with previous research showing that equine PDL cells expressed COL1 and COL3, indicating collagen production and turnover that support remodeling [9]. These proteins are potentially regulated by TGF‐beta, present in ePDLSCs–exo, which stimulates fibroblasts to produce ECM [59–61]. ePDLSCs–EXs thus contribute to tissue remodeling and diabetic wound healing by affecting ECM organization.

## 5. Conclusion

These findings align with evidence that ePDLSCs–EXs have regenerative properties that improve the impaired healing environment of diabetic wounds. They may restore the balance between tissue degradation and regeneration by promoting angiogenesis, modulating inflammation, and supporting ECM deposition. These features suggest that ePDLSCs–EXs may act as paracrine agents, offering stem cell therapeutic benefits without the risks of direct transplantation. Although further molecular studies are needed to clarify the signaling mechanisms, our results demonstrate the potential of ePDLSCs‐derived EXs as a cell‐free therapy for diabetic wounds.

## Author Contributions

Conceptualization was carried out by Medania Purwaningrum. Data were curated by Medania Purwaningrum and Ruchianasari Deliaputri. Formal analysis was conducted by Medania Purwaningrum and Ruchianasari Deliaputri. Funding was secured by Medania Purwaningrum. The investigation involved Medania Purwaningrum, Teguh Budipitojo, and Aris Haryanto. Methodology was developed by Medania Purwaningrum and Ruchianasari Deliaputri. Project administration was managed by Medania Purwaningrum. Resources were provided by Medania Purwaningrum. Software was developed by Medania Purwaningrum. Supervision was handled by Medania Purwaningrum and Teguh Budipitojo. Validation was performed by Medania Purwaningrum, Teguh Budipitojo, and Aris Haryanto. Visualization was created by Medania Purwaningrum and Ruchianasari Deliaputri. The original draft was written by Ruchianasari Deliaputri. Writing, review, and editing were done by Medania Purwaningrum and Ruchianasari Deliaputri.

## Funding

This project was supported by the Department Development Research Grant and Competitive Research Grant from the Faculty of Veterinary Medicine, Universitas Gadjah Mada, Yogyakarta, Indonesia, and the 3rd International Conference on Animal, Veterinary, and Environmental Sciences (3rd ICAVESS 2025) Faculty of Veterinary Medicine, Universitas Gadjah Mada, Yogyakarta, Indonesia.

## Disclosure

All authors have read and approved the final version of the manuscript. The corresponding author had full access to all data in this study and takes full responsibility for the integrity of the data and the accuracy of the data analysis. The lead author affirms that this manuscript is an honest, accurate, and transparent account of the study being reported; that no important aspects of the study have been omitted; and that any discrepancies from the study as planned (and, if relevant, registered) have been explained.

## Conflicts of Interest

The authors declare no conflicts of interest.

## Data Availability

The data supporting the findings of this study are available from the corresponding author upon reasonable request.

## References

[1] Caruso M. , Shuttle S. , Amelse L. , Elkhenany H. , Schumacher J. , and Dhar M. S. , A Pilot Study to Demonstrate the Paracrine Effect of Equine, Adult Allogenic Mesenchymal Stem Cells in Vitro, With a Potential for Healing of Experimentally-Created, Equine Thoracic Wounds in Vivo, Frontiers in Veterinary Science. (2022) 9, 10.3389/fvets.2022.1011905. PMC9702339 36452146

[2] Mund S. J. K. , Kawamura E. , Awang-Junaidi A. H. et al., Homing and Engraftment of Intravenously Administered Equine Cord Blood-Derived Multipotent Mesenchymal Stromal Cells to Surgically Created Cutaneous Wound in Horses: A Pilot Project, Cells. (2020) 9, no. 5, 10.3390/cells9051162. PMC7290349 32397125

[3] Textor J. A. , Clark K. C. , Walker N. J. et al., Allogeneic Stem Cells Alter Gene Expression and Improve Healing of Distal Limb Wounds in Horses, Stem Cells Translational Medicine. (2018) 7, no. 1, 98–108, 10.1002/sctm.17-0071. PMC5746157 29063737

[4] Purbantoro S. D. , Taephatthanasagon T. , Purwaningrum M. et al., Trends of Regenerative Tissue Engineering for Oral and Maxillofacial Reconstruction in Veterinary Medicine, Frontiers in Veterinary Science. (2024) 11, 10.3389/fvets.2024.1325559. PMC10915013 38450027

[5] y Baena A. R. , Casasco A. , and Monti M. , Hypes and Hopes of Stem Cell Therapies in Dentistry: A Review, Stem Cell Reviews and Reports. (2022) 18, no. 4, 1294–1308, 10.1007/s12015-021-10326-4. PMC8748526 35015212

[6] Purwaningrum M. , Jamilah N. S. , Purbantoro S. D. , Sawangmake C. , and Nantavisai S. , Comparative Characteristic Study From Bone Marrow-Derived Mesenchymal Stem Cells, Journal of Veterinary Science. (2021) 22, no. 6, 10.4142/jvs.2021.22.e74. PMC8636658 34697921

[7] Purwaningrum M. , Haryanto A. , Kayanaveda Y. , and Sawangmake C. , Osteogenic Differentiation Potential of Equine Dental Pulp vs. Periodontal Ligament Stem Cells: A Comparative in Vitro Study, Jurnal Medik Veteriner. (2025) 8, no. 2, 262–272, 10.20473/jmv.vol8.iss2.2025.262-272.

[8] Heilen L. B. , Roßgardt J. , Dern-Wieloch J. , Vogelsberg J. , and Staszyk C. , Isolation and Cultivation as Well as in Situ Identification of MSCs From Equine Dental Pulp and Periodontal Ligament, Frontiers in Veterinary Science. (2023) 10, 10.3389/fvets.2023.1116671. PMC10036573 36968463

[9] Mensing N. , Gasse H. , Hambruch N. , Haeger J. D. , Pfarrer C. , and Staszyk C. , Isolation and Characterization of Multipotent Mesenchymal Stromal Cells From the Gingiva and the Periodontal Ligament of the Horse, BMC Veterinary Research. (2011) 7, no. 1, 10.1186/1746-6148-7-42. PMC3161857 21810270

[10] Pöschke A. , Krähling B. , Failing K. , and Staszyk C. , Molecular Characteristics of the Equine Periodontal Ligament, Frontiers in Veterinary Science. (2018) 4, 10.3389/fvets.2017.00235. PMC5768624 29376061

[11] Gunawardena T. N. A. , Rahman M. T. , Abdullah B. J. J. , and Abu Kasim N. H. , Conditioned Media Derived From Mesenchymal Stem Cell Cultures: The next Generation for Regenerative Medicine, Journal of Tissue Engineering and Regenerative Medicine. (2019) 13, no. 4, 569–586, 10.1002/term.2806. 30644175

[12] El Moshy S. , Radwan I. A. , Rady D. et al., Dental Stem Cell-Derived Secretome/Conditioned Medium: The Future for Regenerative Therapeutic Applications, Stem Cells International. (2020) 2020, 1–29, 10.1155/2020/7593402. PMC7013327 32089709

[13] González-González A. , García-Sánchez D. , Dotta M. , Rodríguez-Rey J. C. , and Pérez-Campo F. M. , Mesenchymal Stem Cells Secretome: The Cornerstone of Cell-Free Regenerative Medicine, World Journal of Stem Cells. (2020) 12, 1529–1552, 10.4252/wjsc.v12.i12.1529. PMC7789121 33505599

[14] Bar J. K. , Lis-Nawara A. , and Grelewski P. G. , Dental Pulp Stem Cell-Derived Secretome and Its Regenerative Potential, International Journal of Molecular Sciences. (2021) 22, no. 21, 10.3390/ijms222112018. PMC8584775 34769446

[15] Kou M. , Huang L. , Yang J. et al., Mesenchymal Stem Cell-Derived Extracellular Vesicles for Immunomodulation and Regeneration: A next Generation Therapeutic Tool?, Cell Death and Disease. (2022) 13, no. 7, 10.1038/s41419-022-05034-x. PMC9252569 35787632

[16] Xia E. J. , Zou S. , Zhao X. , Liu W. , Zhang Y. , and Zhao I. S. , Extracellular Vesicles as Therapeutic Tools in Regenerative Dentistry, Stem Cell Research & Therapy. (2024) 15, no. 1, 10.1186/s13287-024-03936-5. PMC11476107 39402576

[17] Arévalo‐Turrubiarte M. , Baratta M. , Ponti G. , Chiaradia E. , and Martignani E. , Extracellular Vesicles From Equine Mesenchymal Stem Cells Decrease Inflammation Markers in Chondrocytes in Vitro, Equine Veterinary Journal. (2022) 54, no. 6, 1133–1143, 10.1111/evj.13537. PMC9787580 34741769

[18] Clarke E. J. , Johnson E. , Caamaño Gutierrez E. et al., Temporal Extracellular Vesicle Protein Changes Following Intraarticular Treatment With Integrin α10β1-Selected Mesenchymal Stem Cells in Equine Osteoarthritis, Frontiers in Veterinary Science. (2022) 9, 10.3389/fvets.2022.1057667. PMC9730043 36504839

[19] Klymiuk M. C. , Balz N. , Elashry M. I. , Heimann M. , Wenisch S. , and Arnhold S. , Exosomes Isolation and Identification From Equine Mesenchymal Stem Cells, BMC Veterinary Research. (2019) 15, no. 1, 10.1186/s12917-019-1789-9. PMC6348641 30691449

[20] Denyer A. L. , O’Neill D. G. , Brodbelt D. C. , Holder A. , Catchpole B. , and Davison L. J. , Epidemiology and Clinical Management of 1072 Dogs With Diabetes Mellitus in a UK Diabetes Register, Companion Animal Health and Genetics. (2025) 12, no. 1, 10.1186/s40575-025-00146-x. PMC12512512 42135839

[21] Waite O. , Gostelow R. , Wright E. , Jepson R. E. , Brodbelt D. C. , and O’Neill D. G. , Frequency, Risk Factors, and Mortality for Diabetes Mellitus in 1 225 130 Cats Under Primary Veterinary Care in the United Kingdom in 2019, Journal of Veterinary Internal Medicine. (2025) 39, no. 4, 10.1111/jvim.70161. PMC12171992 40525629

[22] Durham A. E. , Hughes K. J. , Cottle H. J. , Rendle D. I. , and Boston R. C. , Type 2 Diabetes Mellitus With Pancreatic β Cell Dysfunction in 3 Horses Confirmed With Minimal Model Analysis, Equine Veterinary Journal. (2009) 41, 924–929, 10.2746/042516409X452152. 20383993

[23] Kaczmarek K. , Janicki B. , and Głowska M. , Insulin Resistance in the Horse: A Review, Journal of Applied Animal Research. (2016) 44, no. 1, 424–430, 10.1080/09712119.2015.1091340.

[24] Stefaniuk-Szmukier M. , Piórkowska K. , and Ropka-Molik K. , Equine Metabolic Syndrome: A Complex Disease Influenced by Multifactorial Genetic Factors, Genes. (2023) 14, no. 8, 10.3390/genes14081544. PMC10454496 37628596

[25] Burgess J. L. , Wyant W. A. , Abujamra B. A. , Kirsner R. S. , and Jozic I. , Diabetic Wound-Healing Science, Medicina (Lithuania). (2021) 57, no. 10, 10.3390/medicina57101072. PMC8539411 34684109

[26] Louiselle A. E. , Niemiec S. M. , Zgheib C. , and Liechty K. W. , Macrophage Polarization and Diabetic Wound Healing, Translational Research. (2021) 236, 109–116, 10.1016/j.trsl.2021.05.006. 34089902

[27] Calibo M. B. T. , Treatment of Chronic and Severe Diabetes Mellitus With Ketoacidosis in a Four-Year-Old Intact Female American Pit Bull Terrier, Asian Journal of Research in Animal and Veterinary Sciences. (2024) 7, no. 2, 109–121, 10.9734/ajravs/2024/v7i2291.

[28] Bradley C. W. , Volk S. W. , Brisson R. K. , and Mauldin E. A. , Wounds in a 14‐Year‐Old Cat With Diabetes Mellitus, Veterinary Dermatology. (2020) 31, no. 6, 502–504, 10.1111/vde.12884. 32886372

[29] Putra A. and Plowgian C. , Uncommon Dermatologic Manifestation (Paronychia) in a Cat With Diabetes Mellitus, Topics in Companion Animal Medicine. (2024) 60, 10.1016/j.tcam.2024.100874. 38616020

[30] Morgan R. A. , Keen J. A. , Walker B. R. , and Hadoke P. W. F. , Vascular Dysfunction in Horses With Endocrinopathic Laminitis, PLoS One. (2016) 11, no. 9, 10.1371/journal.pone.0163815. PMC5042533 27684374

[31] Storms N. , Medina Torres C. , Franck T. , Sole Guitart A. , de la Rebière G. , and Serteyn D. , Presence of Myeloperoxidase in Lamellar Tissue of Horses Induced by an Euglycemic Hyperinsulinemic Clamp, Frontiers in Veterinary Science. (2022) 9, 10.3389/fvets.2022.846835. PMC8962398 35359667

[32] Akkus G. and Sert M. , Diabetic Foot Ulcers: A Devastating Complication of Diabetes Mellitus Continues Non-Stop in Spite of New Medical Treatment Modalities, World Journal of Diabetes. (2022) 13, no. 12, 1106–1121, 10.4239/wjd.v13.i12.1106. PMC9791571 36578865

[33] Purwaningrum M. , Giachelli C. M. , Osathanon T. , Rattanapuchpong S. , and Sawangmake C. , Dissecting Specific Wnt Components Governing Osteogenic Differentiation Potential by Human Periodontal Ligament Stem Cells Through Interleukin-6, Scientific Reports. (2023) 13, no. 1, 10.1038/s41598-023-35569-8. PMC10239497 37270571

[34] Sholihah I. A. and Barlian A. , Anti-Inflammatory Potency of Human Wharton’s Jelly Mesenchymal Stem Cell-Derived Exosomes on L2 Cell Line Induced by Lipopolysaccharides, Advanced Pharmaceutical Bulletin. (2024) 14, no. 2, 434–444, 10.34172/apb.2024.027. PMC11347737 39206409

[35] Choi E. W. , Lim I.-R. , Park J. H. , Song J. , Choi B. , and Kim S. , Exosomes Derived From Mesenchymal Stem Cells Primed With Disease-Condition-Serum Improved Therapeutic Efficacy in a Mouse Rheumatoid Arthritis Model via Enhanced TGF-β1 Production, Stem Cell Research & Therapy. (2023) 14, no. 1, 10.1186/s13287-023-03523-0. PMC10552321 37794417

[36] Sormunen A. , Koivulehto E. , Alitalo K. , Saksela K. , Laham-Karam N. , and Ylä-Herttuala S. , Comparison of Automated and Traditional Western Blotting Methods, Methods and Protocols. (2023) 6, no. 2, 10.3390/mps6020043. PMC10142486 37104025

[37] Dyanitha Y. S. , Haryanto A. , Budipitojo T. et al., Canine Dental Pulp Stem Cell-Derived Exosomes: Proteomic Characterization and First Xenogeneic Application for Post-Castration Wound Healing in Cats, Veterinary World. (2026) 19, 3755–3773, 10.14202/vetworld.2026.3755-3773. PMC13590167 42764957

[38] Oyebode O. A. , Jere S. W. , and Houreld N. N. , Current Therapeutic Modalities for the Management of Chronic Diabetic Wounds of the Foot, Journal of Diabetes Research. (2023) 2023, 1–10, 10.1155/2023/1359537. PMC9937766 36818748

[39] Ma Z.-J. , Yang J.-J. , Lu Y.-B. , Liu Z. Y. , and Wang X. X. , Mesenchymal Stem Cell-Derived Exosomes: Toward Cell-Free Therapeutic Strategies in Regenerative Medicine, World Journal of Stem Cells. (2020) 12, no. 8, 814–840, 10.4252/wjsc.v12.i8.814. PMC7477653 32952861

[40] Qian X. , An N. , Ren Y. , Yang C. , Zhang X. , and Li L. , Immunosuppressive Effects of Mesenchymal Stem Cells-Derived Exosomes, Stem Cell Reviews and Reports. (2021) 17, no. 2, 411–427, 10.1007/s12015-020-10040-7. 32935222

[41] Hu J.-C. , Zheng C.-X. , Sui B.-D. , Liu W. J. , and Jin Y. , Mesenchymal Stem Cell-Derived Exosomes: A Novel and Potential Remedy for Cutaneous Wound Healing and Regeneration, World Journal of Stem Cells. (2022) 14, no. 5, 318–329, 10.4252/wjsc.v14.i5.318. PMC9157601 35722196

[42] Jing S. , Li H. , and Xu H. , Mesenchymal Stem Cell Derived Exosomes Therapy in Diabetic Wound Repair, International Journal of Nanomedicine. (2023) 18, 2707–2720, 10.2147/IJN.S411562. PMC10216860 37250470

[43] Liu L. and Liu D. , Bioengineered Mesenchymal Stem Cell-Derived Exosomes: Emerging Strategies for Diabetic Wound Healing, Burns & Trauma. (2024) 12, 10.1093/burnst/tkae030. PMC11250359 39015252

[44] Ostrowska-Podhorodecka Z. , Ding I. , Lee W. et al., Vimentin Tunes Cell Migration on Collagen by Controlling β1 Integrin Activation and Clustering, Journal of Cell Science. (2021) 134, no. 6, 10.1242/jcs.254359. 33558312

[45] Moon C. H. , Ko H. G. , Lee H. et al., Mesenchymal Stem Cell‐Inspired Microneedle Platform for Nir‐Responsive Immunomodulation and Accelerated Chronic Wound Healing, Advanced Materials. (2026) 38, no. 11, 10.1002/adma.202514081. PMC12921356 41137637

[46] Abd‐Rabou A. A. , Youssef A. M. , El‐Khonezy M. I. , Kotob S. E. , and Kishta M. S. , Anti‐Inflammatory/Antioxidant Features of Adipose Tissue Mesenchymal Stem Cells Conditioned Media for Doped TiO_2_ Nanoparticles in Induced Inflammation, ChemistryOpen. (2025) 14, no. 8, 10.1002/open.202500261. PMC12368888 40489075

[47] Atalay M. , Oksala N. , Lappalainen J. , Laaksonen D. , Sen C. , and Roy S. , Heat Shock Proteins in Diabetes and Wound Healing, Current Protein and Peptide Science. (2009) 10, no. 1, 85–95, 10.2174/138920309787315202. PMC2743605 19275675

[48] Jiang X. , Ma J. , Xue K. et al., Highly Bioactive MXene-M2-Exosome Nanocomposites Promote Angiogenic Diabetic Wound Repair Through Reconstructing High Glucose-Derived Immune Inhibition, ACS Nano. (2024) 18, no. 5, 4269–4286, 10.1021/acsnano.3c09721. 38270104

[49] Kim H. , Wang S. Y. , Kwak G. , Yang Y. , Kwon I. C. , and Kim S. H. , Exosome‐Guided Phenotypic Switch of M1 to M2 Macrophages for Cutaneous Wound Healing, Advanced Science. (2019) 6, no. 20, 10.1002/advs.201900513. PMC6794619 31637157

[50] Kishta M. S. , Abd-Rabou A. A. , Sarkissian G. K. et al., Exosome Mediated Delivery of Epigallocatechin 3 Gallate as a Novel Approach to Alleviate Psoriasis Symptoms Through Cytokine and Apoptotic Pathway Modulation, Scientific Reports. (2025) 15, no. 1, 10.1038/s41598-025-10886-2. PMC12357947 40818978

[51] Tutuianu R. , Rosca A.-M. , Iacomi D. M. , Simionescu M. , and Titorencu I. , Human Mesenchymal Stromal Cell-Derived Exosomes Promote in Vitro Wound Healing by Modulating the Biological Properties of Skin Keratinocytes and Fibroblasts and Stimulating Angiogenesis, International Journal of Molecular Sciences. (2021) 22, no. 12, 10.3390/ijms22126239. PMC8228793 34207905

[52] Dallacasagrande V. and Hajjar K. A. , Annexin A2 in Inflammation and Host Defense, Cells. (2020) 9, no. 6, 10.3390/cells9061499. PMC7348701 32575495

[53] Kumar P. , Kumari R. R. , Kumar M. , Kumar S. , and Chakrabarti A. , Current Practices and Research Updates on Diabetes Mellitus in Canine, Veterinary World. (2014) 7, no. 11, 952–959, 10.14202/vetworld.2014.952-959.

[54] Jian K. , Yang C. , Li T. et al., PDGF-BB-Derived Supramolecular Hydrogel for Promoting Skin Wound Healing, Journal of Nanobiotechnology. (2022) 20, no. 1, 10.1186/s12951-022-01390-0. PMC9044828 35473604

[55] Simurda T. , Brunclikova M. , Asselta R. et al., Genetic Variants in the FGB and FGG Genes Mapping in the Beta and Gamma Nodules of the Fibrinogen Molecule in Congenital Quantitative Fibrinogen Disorders Associated With a Thrombotic Phenotype, International Journal of Molecular Sciences. (2020) 21, no. 13, 10.3390/ijms21134616. PMC7369898 32610551

[56] Zuliani-Alvarez L. and Midwood K. S. , Fibrinogen-Related Proteins in Tissue Repair: How a Unique Domain With a Common Structure Controls Diverse Aspects of Wound Healing, Advances in Wound Care. (2015) 4, no. 5, 273–285, 10.1089/wound.2014.0599. PMC4432967 26005593

[57] Bundgaard L. , Jacobsen S. , Sørensen M. A. et al., The Equine Peptideatlas: A Resource for Developing Proteomics‐Based Veterinary Research, Proteomics. (2014) 14, no. 6, 763–773, 10.1002/pmic.201300398. PMC4340707 24436130

[58] Mathew-Steiner S. S. , Roy S. , and Sen C. K. , Collagen in Wound Healing, Bioengineering. (2021) 8, no. 5, 10.3390/bioengineering8050063. PMC8151502 34064689

[59] Zhao B. , Zhang X. , Zhang Y. et al., Human Exosomes Accelerate Cutaneous Wound Healing by Promoting Collagen Synthesis in a Diabetic Mouse Model, Stem Cells and Development. (2021) 30, no. 18, 922–933, 10.1089/scd.2021.0100. 34167333

[60] Marconi G. D. , Fonticoli L. , Rajan T. S. et al., Transforming Growth Factor-Beta1 and Human Gingival Fibroblast-to-Myofibroblast Differentiation: Molecular and Morphological Modifications, Frontiers in Physiology. (2021) 12, 10.3389/fphys.2021.676512. PMC8176099 34093237

[61] Valluru M. , Staton C. A. , Reed M. W. R. , and Brown N. J. , Transforming Growth Factor-? and Endoglin Signaling Orchestrate Wound Healing, Frontiers in Physiology. (2011) 2, 10.3389/fphys.2011.00089. PMC3230065 22164144

